# Synthesis of some new Thieno[2,3-*b*]pyridines, Pyrimidino[4',5':4,5]thieno[2,3-*b*]pyridine and Pyridines Incorporating 5-Bromobenzofuran-2-yl Moiety

**DOI:** 10.3390/molecules20010822

**Published:** 2015-01-07

**Authors:** Nadia Abdelhamed Abdelriheem, Sayed Abdel-Kader Ahmad, Abdou Osman Abdelhamid

**Affiliations:** 1 Department of Chemistry, Faculty of Science, Cairo University, Giza 12613, Egypt; E-Mail: nadia.abdelhamid5@gmail.com; 2 Department of Chemistry, Faculty of Science, Beni-Suef University, Beni-Suef 62514, Egypt; E-Mail: Abdelhamid45@gmail.com

**Keywords:** thieno[2,3-*b*]pyridines, pyrimidino[4',5':4,5]thieno[2,3-*b*]pyridine, pyridines, 5-bromobenzofuran, urea, carbamate

## Abstract

2-Sulfanyl-6-(2-thienyl)pyridine-3-carbonitrile, 1-Amino-6-(5-bromo-benzofuran-2-yl)-2-oxo-1,2-dihydro-pyridine-3-carbonitrile, thieno[2,3-*b*]pyridins, pyrimidino[4',5':4,5]thieno[2,3-*b*]pyridine, quinazoline and carbamate derivatives were synthesized from sodium 3-(5-bromobenzofuran-2-yl)-3-oxoprop-1-en-1-olate with. The newly synthesized compounds were elucidated by elemental analysis, spectral data, and alternative synthesis whenever possible and chemical transportation.

## 1. Introduction

The thieno[2,3-*b*]pyridine derivatives occupy special place and have attracted considerable attention because of their broad pharmacological activities, including anticancer [1,2,3,4,5,6,7,8,9], antiviral [10,11,12,13], anti-inflammatory [14,15,16,17], antimicrobial [18,19], antidiabetic [20,21,22,23], antihypertensive [24,25,26] and osteogenic [27,28] activities, in addition to treatment of CNS disorders [29,30,31]. Also, pyridine derivatives of different heterocyclic nucleus have shown potent pharmacological properties like antifungal [32,33], antitubercular [34], antibacterial [35], antimicrobial [36], insecticida [37]*.* In view of these findings and in continuation to our previous work [38,39,40,41,42,43], we report here the convenient synthesis of some new thieno[2,3-*b*]pyridines, pyrimidino[4',5':4,5]thieno[2,3-*b*]pyridines and pyridines incorporating 5-bromobenzofuran-2-yl moiety.

## 2. Results and Discussion

**Scheme 1:**
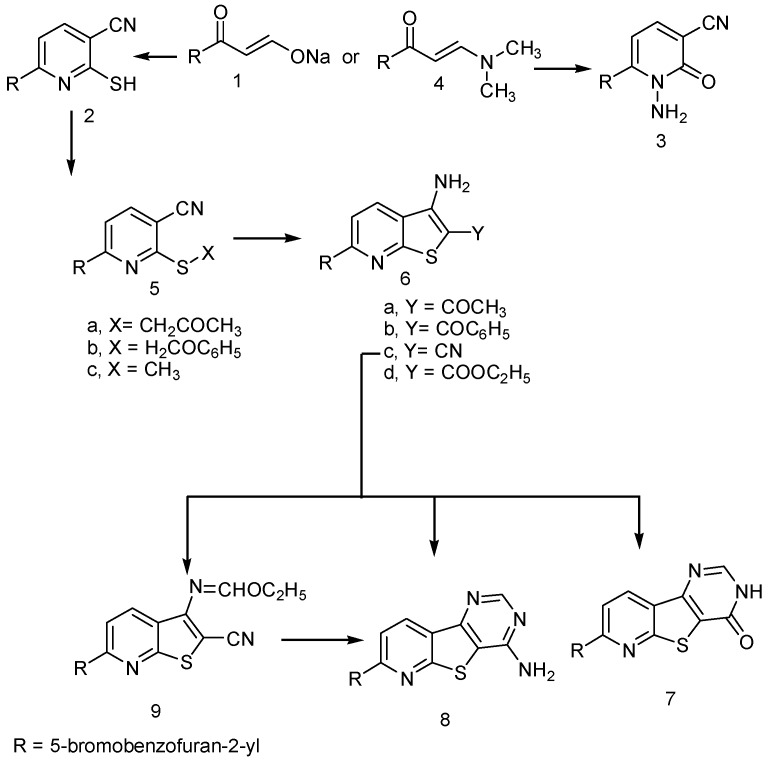
Synthesis of pyridenes **2**, **3**, thieno[2,3-*b*]pyridenes **6a**–**d** and pyrimidine[4',5':4,5]thieno[2,3-*b*]pyridines **7** and **8**.

Treatment of sodium 3-(5-bromobenzofuran-2-yl)-3-oxoprop-1-en-1-olate (**1**) [44] with each of cyanothioacetamide or 2-cyanoacetohydrazide in piperidinium acetate under refluxed to give 6-(5-bromobenzofuran-2-yl)-2-thioxo-1,2-dihydropyridine-3-carbonitrile (**2**) and 1-amino-6-(5-bromobenzofuran-2-yl)-2-oxo-1,2-dihydro-pyridine-3-carbonitrile (**3**), respectively in a good yield (Scheme 1). Structure **2** was elucidated by elemental analysis, spectra, and chemical transformation. 6-(5-bromobenzofuran-2-yl)-2-thioxo-1,2-dihydropyridine-3-carbonitrile (**2**) was reacted with chloroacetone in *N,N*-dimethylformamide containing potassium hydroxide to afford the product corresponding to addition and dehydrochlorination reactions. The IR spectrum of this product showed bands at 2218 and 1700 cm^−1^ corresponding to CN and CO groups. Its ^1^H-NMR spectrum revealed the signals at δ 2.39 (s, 3H, CH_3_), 4.38 (s, 2H, SCH_2_) and 7.23–7.97 (m, 6H, ArH’s). Based on these data, these reaction products could be formulated as 2-(2-oxopropylthio)-6-(5-bromobenzofuran-2-yl)pyridine-3-carbonitrile (**5a**). Further confirmation of the structure of **5a** arose from their cyclization in boiling ethanol containing a catalytic amount of piperidine to give the corresponding 1-(3-amino-6-(5-bromobenzofuran-2-yl)thieno[2,3-*b*]pyridin-2-yl)ethanone (**6a**) (Scheme 1). The IR spectrum of **6a** showed no band of the CN function but the bands at 3274, 3174 (NH_2_ group). ^1^H-NMR spectrum of **6a** revealed an absence of signals of the -SCH_2_- group and the presence of the NH_2_ protons. These findings proved that the CN and the -SCH_2_- groups were both involved in the cyclization step leading to **6a**.

Also, **2** was reacted with each ω-bromoacetophenone and idomethane in *N,N*-dimethylformamide containing potassium hydroxide to afford 6-(5-bromo-benzofuran-2-yl)-2-(2-oxo-2-phenyl-ethylsulfanyl)-nicotinonitrile (**5b**) and 6-(5-bromobenzofuran-2-yl)-2-(methylthio)nicotinonitrile. Compound **5b** was converted to (3-amino-6-(5-bromobenzofuran-2-yl)thieno[2,3-*b*]pyridin-2-yl)(phenyl)methanone (**6b**) by its boiling in ethanolic piperidine solution. ^1^H-NMR of **6b** showed signals at δ 4.05 (s, 2H, NH_2_), and 7.14–7.78 (m, 11H, ArH’s) (Scheme 1).

In contrast, compound **2** was reacted with each of chloroacetonitrile and ethyl chloroacetate afforded 3-amino-6-(5-bromobenzofuran-2-yl)thieno[2,3-*b*]pyridine-2-carbonitrile (**6c**) and ethyl 3-amino-6-(5-bromobenzofuran-2-yl)thieno[2,3-*b*]pyridine-2-carboxylate (**6d**), in a good yield. Structure of **6c** was confirmed by elemental analysis, spectral data and chemical transportation. Thus, compound **6c** was reacted with each of formic acid or formamide to give the corresponding 7-(2-thienyl)-3-hydropyrimidino[4',5':4,5]thieno[2,3-*b*]pyridine-4-one (**7**) and 7-(2-thienyl)pyrimidine[4',5':4,5]thieno[2,3-*b*]pyridine-4-ylamine (**8**), respectively (Scheme 1). Structures **7** and **8** were established on the basis of spectral data and elemental analysis. Thus, IR spectrum of **7** revealed a band at 1666 (CO). IR spectrum of **8** revealed bands at 3320, 3151 (NH_2_). Meanwhile, **6c** reacted with triethyl ortho-formate to give ethyl *N*-[6-(5-bromo-benzofuran-2-yl)-2-cyano-thieno[2,3-*b*]pyridin-3-yl]-formimidoate (**9**). The latter compound was reacted with ammonia or formamide gave a product identical in all aspects (mp., mixed mp., and spectra) with compound **8**.

**Scheme 2:**
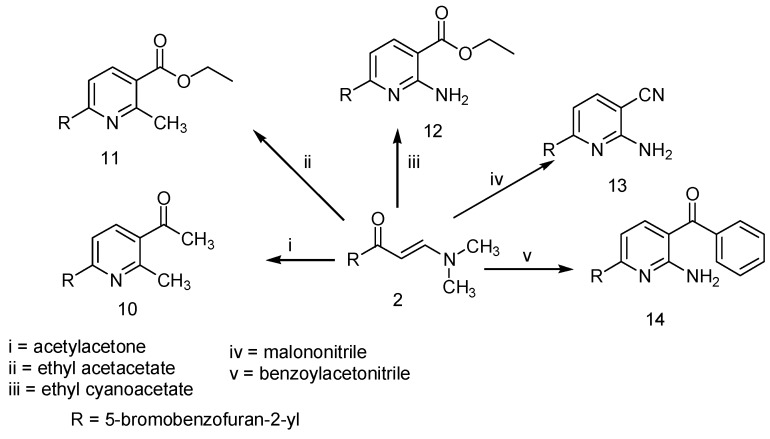
Synthesis of pyridines **10**–**14**.

Treatment of **2** with 2,4-pentanedione, ethyl 3-oxobutanoate, ethyl cyanoactate, malononitrile or benzoylacetonitrile in boiling acetic acid and ammonium acetate under refluxed gave 1-(6-(5-bromobenzofuran-2-yl)-2-methylpyridin-3-yl)ethanone (**10**) and ethyl 6-(5-bromobenzofuran-2-yl)-2-methylpyridine-3-carboxylate (**11**), ethyl 2-amino-6-(5-bromobenzofuran-2-yl)pyridine-3-carboxylate (**12**), 2-amino-6-(5-bromobenzofuran-2-yl)pyridine-3-carbonitrile (**13**) and 2-amino-6-(5-bromobenzofuran-2-yl)-3-benzoylpyridine (**14**), respectively (Scheme 2).

**Scheme 3:**
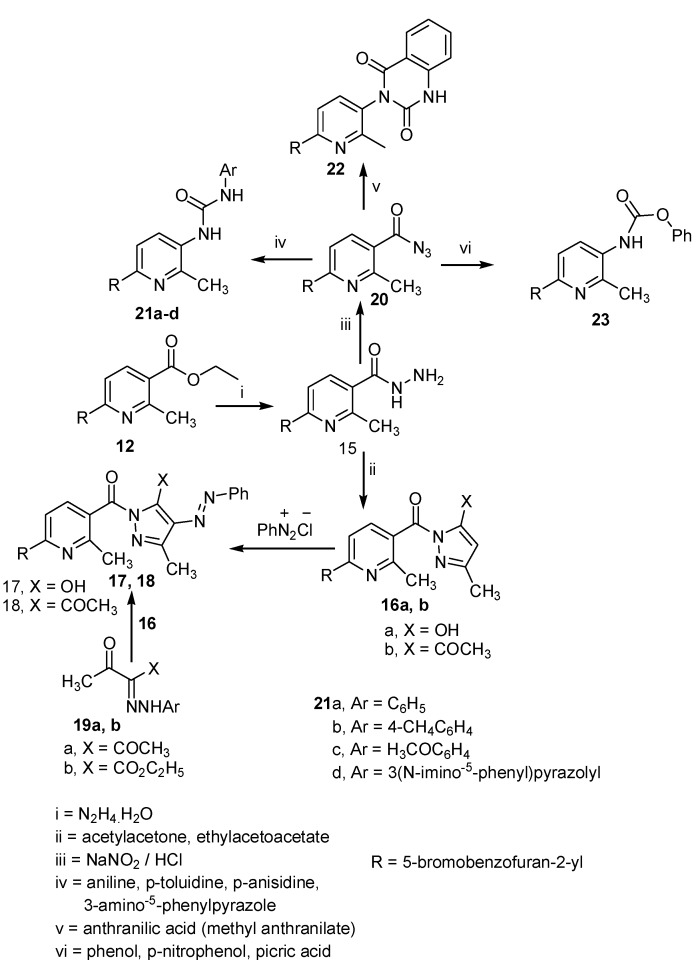
Synthesis of pyridines **15**–**18**, **20**–**22**, quinazoline **22** and carbamates **23**.

Next, Compounds **11** was reacted with hydrazine hydrate afforded 2-methyl-6-(2-oxo-2*H*-chromen-3-yl)pyridine-3-carbohydrazide (**15**). The structure of **15** was elucidated by elemental analyses, spectra and chemical transformations. Thus, compounds **15** was reacted with each of ethyl acetoacetate, acetylacetone and nitrous acid, gave 2-[6-(5-bromo-benzofuran-2-yl)-2-methyl-pyridine-3-carbonyl]-5-methyl-2,4-dihydropyrazol-3-one (**16a**), [6-(5-bromo-benzofuran-2-yl)-2-methyl-pyridin-3-yl]-(3,5-dimethyl-pyrazol-1-yl)-methanone (**16b**) and 6-(5-bromobenzofuran-2-yl)-2-methylnicotinoyl azide (**20**), respectively (Scheme 3).

Structures **16a**, **16b** and **20** were confirmed by elemental analyses, spectral data and chemical transformations. Thus, treatment of **16a** and **16b** with benzenediazonium chloride in ethanolic sodium acetate gave **17** and **18**, respectively. Structures **17** and **18** were confirmed by elemental analyses, spectral data and alternative synthetic route (reaction of the appropriate ethyl 3-oxo-2-(2-phenylhydrazono)butanoate (**19a**) [45] or 3-(2-phenylhydrazono)pentane-2,4-dione (**19b**) [46] with **15** in boiling acetic acid under refluxed gave identical product in aspects (mp., mixed mp. and spectra) with corresponding compounds **17** and **18**). structure **20** was established by elemental analyses, spectral and chemical transformation. Thus, treatment of **20** with each of the appropriate aromatic amine (aniline, p-toluidine, p-anisidine, 3-amino-5-phenylpyrazole or anthranilic acid (or methyl anthranilate) in boiling dioxane and phenol in boiling benzene gave 1-[6-(5-bromo-benzofuran-2-yl)-2-methyl-pyridin-3-yl]-3-substituted urea **21a**–**d**, 3-[6-(5-bromo-benzofuran-2-yl)-2-methylpyridin-3-yl]-1*H*-quinazoline-2,4-dione (**22**) and phenyl [6-(5-bromo-benzofuran-2-yl)-2-methyl-pyridin-3-yl]-carbamoate (**23**) (Scheme 3). Structures **21**–**23** were elucidated by elemental analyses and spectral data.

## 3. Experimental Section

All melting points were determined on an Electrothermal melting point apparatus and are uncorrected. IR spectra were recorded (KBr discs) on a Shimadzu FT-IR 8201 PC spectrophotometer (Kyoto, Japan). ^1^H-NMR and ^13^C-NMR spectra were recorded in CDCl_3_ and (CD_3_)_2_SO solutions on a Varian Gemini 300 MHz (Varian Inc., Palo Alto, CA, USA) and JNM-LA 400 FT-NMR system spectrometer (Japan Electronic Optics Laboratory Co. Ltd., Tokyo, Japan) and chemical shifts are expressed in δ units using TMS as internal reference. Mass spectra were recorded on a Shimadzu GCMS-QP1000 EX mass spectrometer (70 eV, Shimadzu, Kyoto, Japan). Elemental analyses were carried out at Micro analytical Center of the University of Cairo, Giza, Egypt.

### 3.1. General Procedure for the Synthesis of 6-(5-Bromobenzofuran-2-yl)-2-thioxo-1,2-dihydropyridine-3-carbonitrile *(**2**)* and 1-Amino-6-(5-bromobenzofuran-2-yl)-2-oxo-1,2-dihydropyridine-3-carbonitrile *(**3**)*

***Method A***: A mixture of sodium 3-(5-bromobenzofuran-2-yl)-3-oxoprop-1-en-1-olate (**1**) (1.43 g, 5 mmol), the appropriate cyanothioacetamide or 2-cyanoacetohydrazide (5 mmol), and few catalytic drops of acetic acid was thoroughly ground with a pestle in an open mortar at room temperature for 3–5 min until the mixture turned into a melt. Grinding of the initial syrup was continued for 5–10 min, and the reaction was monitored by TLC. The solid was washed with water and recrystallized from the appropriate solvent gave the corresponding fused pyridines **2** and **3**, respectively.

***Method B***: A mixture of sodium 3-(5-bromobenzofuran-2-yl)-3-oxoprop-1-en-1-olate (**1**) (1.43 g, 5 mmol) and the appropriate cyanothioacetamide or 2-cyanoacetohydrazide (5 mmol) in a solution of piperidinum acetate (piperidine (2.5 mL), water (5 mL), and acetic acid (2 mL)) was heated under reflux for about 10 min; acetic acid (1.5 mL) was added to the reaction mixture while boiling. Then the mixture was cooled, and the resulting solid was collected and recrystallized from the appropriate solvent gave **2** and **3**, respectively.

***Method C***: A mixture of 1-(5-bromobenzofuran-2-yl)-3-(dimethylamino)prop-2-en-1-one (**4**) (1.47 g, 5 mmol) and the appropriate cyanothioacetamide or 2- cyanoacetohydrazide (5 mmol) in a solution of ethanol containing catalytical amount of piperdine (20 mL) was refluxed for 4–5 h. The resulting solid was collected and recrystallized to give identical in all aspects (mp., mixed mp. and spectra) with **2** and **3**, respectively.

*6-(5-Bromobenzofuran-2-yl)-2-thioxo-1,2-dihydropyridine-3-carbonitrile* (**2**)*.* Deep red crystals. Yield: 65%, melting point: 172–174 °C (acetic acid). IR (KBr, cm^−1^): 3380 (NH), 3082 (CH), 2218 (CN), 1635 (C=N), 1570 (C=C); ^1^H*-*NMR (400 MHz, DMSO-*d*_6_): δ = 6.97 (s, 1H, furan H-3), 7.28–7.66 (m, 4H, ArH’s), 7.89–7.61 (d, 1H*, J* = 8.0 Hz, ArH’s), 14.42 (s, br., 1H, NH); ^13^C*-*NMR (400 MHz, DMSO-*d*_6_): δ = 101.5 (C18), 102.9 (C9), 114.1 (C1), 115.9 (C14), 116.5 (C16), 117.4 (C5), 122.3 (C2), 129.3 (C13), 129.5 (C15), 144.2 (C6), 148.7 (C8), 154.6 (C11), 159.1 (C8), 182.1 (C4); (CMS, *m/z*, (%); Calcd. for C_14_H_7_BrN_2_OS (331.19) C, 50.77; H, 2.13; Br, 24.13; N, 8.46; S, 9.68 Found: C, 50.66; H, 2.18; Br, 24.07; N, 8.41; S, 9.75.

*l-Amino-6-(5-bromo-benzofuran-2-yl)-2-oxo-1,2-dihydro-pyridine-3-carbonitrile* (**3**)*.* Yellow crystals. Yield: 62%, melting point: > 300 °C (acetic acid). IR (KBr, cm^−1^): 3380,3260 (NH_2_), 3082 (CH), 2218 (CN), 1635 (C=N), 1570(C=C); ^1^H-NMR (400 MHz, DMSO-*d*_6_): δ = 6.12 (s, br., 2H, NH_2_), 7.01 (s, 1H, benzofuran H-3), 7.32–8.17 (m, 5H, ArH’s); ^13^C*-*NMR (400 MHz, DMSO-*d*_6_): δ = 102.5 (C2), 104.7 (C9), 109.8 (C19), 114.1 (C16), 115.5 (C1), 116.5 (C14), 124.3 (C2), 125.4 (C13), 129.3 (C10), 129.57 (C1), 144.8 (C8), 152.1 (C6), 154.0 (C11), 164.4 (C4); Calcd. for C_1__4_H_8_BrN_3_O_2_ (330.14) C, 50.93; H, 2.44; Br, 24.20; N, 12.73 Found: C, 50.88; H, 2.51; Br, 24.11; N, 12.65%.

### 3.2. General Procedure for the Synthesis of 6-(5-bromobenzofuran-2-yl)-2-((2-oxopropyl)thio)-nicotinonitrile *(**5a**)*, 6-(5-bromobenzofuran-2-yl)-2-(2-oxo-2-phenyl-ethylsulfanyl)-nicotinonitrile *(**5b**)* and 6-(5-bromobenzofuran-2-yl)-2-(methylthio)nicotinonitrile *(**5c**)*

***Grinding Method***: Equimolar amounts of **2** (1.66 g, 5 mmol) and potassium hydroxide (0.28 g, 5 mmol) was ground with a pestle in an open mortar followed by the appropriate chloroacetone, ω-bromoacetophenone, or iodomethane (5 mmol) at room temperature for 2–3 min. until the mixture turned into a melt. The initial syrupy reaction mixture solidified within 3–5 min. Grinding was continued for 5–10 min. while the reaction was monitored by TLC. The solid was washed with water and recrystallized from *N,N*-dimethylformamide afforded the corresponding **5a**–**c**, respectively.

***Traditional Method***: A mixture of 6-(5-bromobenzofuran-2-yl)-2-mercaptonicotinonitrile (**2**) (1.66 g, 5 mmol) and potassium hydroxide (0.56 g, 5 mmol) in *N,N*-dimethylformamide (20 mL) was stirred for 2 h. The appropriate chloroacetone, ω-bromoacetophenone or iodomethane (5 mmol) was added to the above mixture. Then, the reaction was stirred for 2 h. The resulting solid was formed after dilution of water was collected and recrystallized from the proper solvent gave pyridine derivatives **5a**–**c**, respectively.

*6-(5-Bromobenzofuran-2-yl)-2-((2-oxopropyl)thio)nicotinonitrile* (**5a**). Brown crystals. Yield: 84%, melting point: 264–266 °C (dioxane). IR (KBr, cm^−1^): 3082 (CH), 2233 (CN), 1700 (CO), 1605 (C=N), 1570 (C=C); ^1^H-NMR (400 MHz, DMSO-*d*_6_): δ = 2.39 (s, 3H, CH_3_), 4.38 (s, 2H, CH_2_), 7.23–7.97 (m, 6H, ArH’s); ^13^C*-*NMR (400 MHz, DMSO-*d*_6_): δ = 19.3 (C 12), 39.8 (C9), 102.8 (C13), 103.1 (C5), 114.1 (C20), 116.2 (C22), 116.5 (C18), 119.1 (C1), 125.3 (17), 129.3 (C14), 129.5 (C19), 136.1 (C6), 150.2 (C2), 154.0 (C15), 159.2 (C4), 159.6 (C8), 201.8 (C1); Calcd. for C_17_H_11_BrN_2_O_2_S (387.25) C, 52.73; H, 2.86; Br, 20.63; N, 7.23; S, 8.28 Found: C, 52.67; H, 2.91; Br, 20.52; N, 7.15; S, 8.10%.

*6-(5-Bromobenzofuran-2-yl)-2-((2-oxo-2-phenylethyl)thio)nicotinonitrile* (**5b**). Deep red crystals. Yield: 80%, melting point: 184–186 °C (acetic acid). IR (KBr, cm^−1^): 3058 (CH), 2221 (CN), 1697 (CO), 1655 (C=N), 1527 (C=C); **^1^**H-NMR (400 MHz, DMSO-*d*_6_): δ = 4.58 (s, 2H, CH_2_), 7.23–8.00 (m, 11H, ArH’s); ^13^C*-*NMR (400 MHz, DMSO-*d*_6_): 37.1 (C18), 102.5 (C8), 103.1 (C5), 114.1 (C15), 116.2 (C19), 116.6 (C13), 119.0 (C1), 125.3 (C12), 128.5 (C24 & C28), 128.7 (C25 & C27), 129.3 (C9), 129.5 (C14), 133.1 (C26), 123.3 (C23), 136.0 (C6), 150.5 (C2), 154.0 (C10), 159.2 (C4), 159.6 (C7), 193.8 (C21), Calcd. For C_22_H_13_BrN_2_O_2_S (449.32) C, 58.81; H, 2.92; Br, 17.78; N, 6.23; S, 7.14 Found: C, 58.92; H, 2.87; Br, 17.84; N, 6.31; S, 7.00%.

*6-(5-Bromobenzofuran-2-yl)-2-(methylthio)pyridine-3-carbonitrile* (**5c**). Brown crystals. Yield: 73%, melting point: 228–230 °C (dioxane). IR (KBr, cm^−1^): 3008 (CH), 2118 (CN), 1642 (C=O), 1566 (C=C); ^1^H-NMR (400 MHz, DMSO-*d*_6_): δ = 2.65 (s, 2H, CH_2_), 7.21–7.89 (m, 6H, ArH’s); ^13^C-NMR (400 MHz, DMSO-*d*_6_): δ = 13.3 (C18), 102.1 (C8), 104.7 (C5), 114.1 (C15), 114.6 (C13), 116.5 (C13), 118.7 (C1), 125.3 (C12), 129.3 (C9), 129.5 (C14), 135.6 (C6), 150.1 (C2), 154.0 (C10), 159.6 (C7), 161.2 (C4); Calcd. for C_15_H_9_BrN_2_OS (345.21) C, 52.19; H, 2.63; Br, 23.15; N, 8.11; S, 9.29 Found: C, 52.00; H, 2.57; Br, 23.08; N, 8.00; 8,9.35%.

### 3.3. General Procedure for the Synthesis of 1-(3-amino-6-(5-bromobenzofuran-2-yl)thieno[2,3-b]pyridin-2-yl)ethan-1-one *(**6a**)*, (3-amino-6-(5-bromobenzofuran-2-yl)thieno[2,3-b]pyridin-2-yl)(phenyl)methanone *(**6b**)*, 3-amino-6-(5-bromobenzofuran-2-yl)thieno[2,3-b]pyridine-2-carbonitrile *(**6c**)* and ethyl 3-Amino-6-(5-bromobenzofuran-2-yl)thieno[2,3-b]pyridine-2-carboxylate *(**6d**)*

***Method A***: A mixture of **2** (1.66 g, 5 mmol) and potassium hydroxide (0.28 g, 5 mmol) in *N,N*dimethylformamide (10 mL) was stirred for 2 h at room temperature. The appropriate of chloroacetone, ω-bromoacetophenone, chloroacetonitrile or ethyl chloroacetate (10 mmol) was refluxed while stirring for 2 h. The resulting solid formed after cooling and dilution with water was collected and crystallized from *N, N*-dimethylformamide afforded **6a**–**d**, respectively.

***Method B***: A mixture of the appropriate **5a** or **5b** (5 mmol) in ethanol (15 mL) and piperidine (5 drops) was heated under refluxed for 2 h. The solid formed was collected and recrystallized gave products identical in all aspects (mp., mixed mp. and spectra) with **6a** and **6b** which were obtained from method A.

*1-(3-Amino-6-(5-bromobenzofuran-2-yl)thieno[2,3-b]pyridin-2-yl)ethanone* (**6a**)*.* Brown crystals. Yield: 84%, melting point: 279–281 °C (dioxane). IR (KBr, cm^−1^): 3274, 3174 (NH_2_), 3074 (CH), 1670 (CO), 1604 (C=N), 1570 (C=C); ^1^H-NMR (400 MHz, DMSO-*d*_6_): δ = 2.36 (s, 2H, CH_3_), 6.90 (s, br., 2H, NH_2_), 7.52–8.70 (m, 6H, ArH’s); ^13^C*-*NMR (400 MHz, DMSO-*d*_6_): δ = 28.5 (C23), 102.8 (C13), 114.1 (C20), 116.5 (C18), 118.3 (C1), 122.3 (C5), 123.8 (C9), 125.3 (C17), 126.5 (C6), 129.3 (C14), 129.5 (C19), 136.0 (C10), 149.5 (C2), 153.8 (C15), 159.4 (C7), 160.0 (C4), 193.2 (C12). Calcd. for C_17_H_11_BrN_2_O_2_S (387.25) C, 52.73; H, 2.86; Br, 20.63; N, 7.23; S, 8.28 Found: C, 52.67; H, 2.78; Br, 20.58; N, 7.11; S, 8.348%.

*[3-Amino-6-(5-bromobenzofuran-2-yl)-thieno[2,3-b]pyridin-2-yl]-phenyl-methanone* (**6b**)*.* Brown crystals. Yield: 84%, melting point: 260–262 °C (dioxane). IR (KBr, cm^−1^): 3425, 3294, 3132 (NH_2_), 3070 (CH), 1672 (CO), 1593 (C=C); ^1^H-NMR (400 MHz, DMSO-*d*_6_): δ = 6.80 (s, br., 2H, NH_2_), 7.44–8.28 (m, 11H, ArH’s); Calcd. for C_22_H_13_BrN_2_O_2_S (449.32) C, 58.81; H, 2.92; Br, 17.78; N, 6.23; S, 7.14 Found: C, 58.75; H, 3.01; Br, 17.84; N, 6.32; S, 7.00%.

*2-(3-Amino-6-(5-bromobenzofuran-2-yl)thieno[2,3-b]pyridin-2-yl)-2-carbonitrile* (**6c**). Brown crystals. Yield: 90%, melting point: 280–282 °C (dioxane). IR (KBr, cm^−1^): 3425, 3348, 3247 (NH_2_), 3070 (CH), 2194 (CN), 1658 (C=N), 1569 (C=C); ^1^H-NMR (400 MHz, DMSO-*d*_6_): δ = 7.39–8.62 (m, 8H, ArH’s and NH_2_); Calcd. for C_16_H_8_BrN_3_OS (370.22) C, 51.91; H, 2.18; Br, 21.58; N, 11.35; S, 8.66 Found: C, 52.01; H, 2.22; Br, 21.51; N, 11.39; 8,8.59%.

*Ethyl 3-amino-6-(5-bromobenzofuran-2-yl)thieno[2,3-b]pyridine-2-carboxylate* (**6d**). Yellow crystals. Yield: 87%, melting point: 290–292 °C (dioxan). IR (KBr, cm^−1^): 3293, 3197 (NH_2_), 2979 (CH), 1670 (CO), 1611 (C=N), 1556 (C=C); ^1^H-NMR (400 MHz, DMSO-*d*_6_): δ = 1.30 (t, 3H, *J = 7.5 Hz*, CH_2_CH_3_), 4.27 (q, 2H, *J = 7.5 Hz*, CH_2_CH_3_), 7.34–8.64 (m, 8H, ArH’s and NH_2_); ^13^C*-*NMR (400 MHz, DMSO-*d*_6_): δ = 8.3 (C24), 33.7 (C23), 102.7 (C13), 114.2 (C20), 116.5 ( C18), 118.6 (C1), 121.5 (C5), 125.0 (C17), 126.3 (C9), 126.7 (C6), 129.3 (C14), 129.6 (C19), 135.4 (C10), 149.5 (C2), 154.0 (C15), 159.6 (C7), 160.0 (C4), 198.5 (C12). Calcd. for C_18_H_13_BrN_2_O_3_S (417.28) C, 51.81; H, 3.14; Br, 19.15; N, 6.71; S, 7.68 Found: C, 51.92; H, 3.24; Br, 19.00; N, 6.61; 8,7.72%.

### 3.4. Synthesis of 7-(5-bromobenzofuran-2-yl)pyrido[3',2':4,5]thieno[3,2-d]pyrimidin-4(3H)-one *(**7**)*, 7-(5-bromobenzofuran-2-yl)pyrido[3',2':4,5]thieno[3,2-d]pyrimidin-4-amine *(**8**)* and ethyl (E)-N-(6-(5-Bromobenzofuran-2-yl)-2-cyanothieno[2,3-*b*]pyridin-3-yl)formimidate *(**9**)*

*7-(5-bromobenzofuran-2-yl)pyrido[3',2':4,5]thieno[3,2-d]pyrimidin-4(3H)-one* (**7**)*.* A mixture of **6c** (1.85 g, 5 mmol) and formic acid (7 mL, 99%) in *N,N,-*dimethylformamide (5 mL) was boiled under reflux for 7 h. The reaction mixture was poured onto ice (30 g). The solid so formed was collected and recrystallized from DMF gave 7 as brown crystals. Yield: 72%, melting point: > 300 °C (DMF). IR (KBr, cm^−1^): 3320 (NH), 3001 (CH), 1666 (CO), 1569 (C=C); ^1^H-NMR (400 MHz, DMSO-*d*_6_): δ = 7.01–8.21 (m, 7H, ArH’s), 12.85 (s, br., 1H, NH); MS, *m/z*, (%): 399 (M+1, 29%), 398 (M^+^, 100%), 397 (M−1,12%), 371 (17%), 370 (67%), 200 (7%), 199 (7%), 105 (10%), 77 (27%); Calcd. for C_17_H_8_BrN_3_O2S (398.23) C, 51.27; H, 2.02; Br, 20.06; N, 10.55; S, 8.05 Found: C, 51.15; H, 1.95; Br, 20.00; N, 10.42; S, 7.87%.

*7-(5-bromobenzofuran-2-yl)pyrido[3',2':4,5]thieno[3,2-d]pyrimidin-4-amine* (**8**). ***Method A***: A mixture of **6c** (1.85 g, 5 mmol) and formamide (5 mL, 99%) in *N,N,-*dimethylformarnide (5 mL) was boiled under reflux for 7 h. The reaction mixture was poured onto ice (30 g). recrystallized from DMF to give **8** as brown crystals. Yield: 78%, melting point: > 300 °C. The solid so formed was collected and (DMF). IR (KBr, cm^−1^): 3320, 3151 (NH_2_), 3001 (CH), 1648 (C=N), 1573 (C=C); ^1^H-NMR (400 MHz, DMSO-*d*_6_): δ = 6.88 (s, br., 2H, NH_2_), 7.31–8.11 (m, 7H, ArH’s); MS, *m/z* (%): 399 (M+2, 29%), 398 (M+1, 100%), 397 (M^+^,12%), 371 (17%), 370 (67%), 200 (7%), 199 (7%), 105 (10%), 77 (27%); Calcd. for C_17_H_9_BrN_4_OS (397.25) C, 51.40; H, 2.28; Br, 20.11; N, 14.10; S, 8.07 Found: C, 51.31; H, 2.32; Br, 20.00; N, 14.23; S, 7.88%. ***Method B***: A mixture of ethyl *N*-[6-(5-bromo-benzofuran-2-yl)-2-cyano-thieno[2,3-*b*]pyridin-3-yl]-formimidoate (**9**) (0.5 g) and formamide (0.5 mL) in *N,N,-*dimethylformamide (5 mL) was boiled for 2 h. The solid so formed was collected and recrystallized from DMF gave a product identical in all aspects (mp., mixed mp. and spectra) with product **8**.

*Ethyl N-[6-(5-bromobenzofuran-2-yl)-2-cyano-thieno[2,3-b]pyridin-3-yl]-formimidoate* (**9**). A mixture of **2d** (1.85 g, 5 mmol) and triethyl ortho-formate (1.48 g, 10 mmol) in acetic anhydride (20 mL) was heated under reflux for 6 h. The reaction mixture was poured onto ice (30 g). The resulting solid was collected and recrystallized from dioxane gave **9** as brown crystals. Yield: 71%, melting point: 250–252 °C (dioxane). IR (KBr, cm^−1^): 3070 (CH), 2194 (CN), 1648 (C=N), 1573 (OC); ^1^H-NMR (400 MHz, DMSO-*d*_6_): δ = 1.37 (t, 3H, *J* = 8.0 Hz, CH_2_CH_3_), 4.32 (q, 2H, *J* = 8.0 Hz, CH_2_CH_3_), 7.31–8.22 (m, 7H, ArH’s and CH=); ^13^C*-*NMR (400 MHz, DMSO-*d*_6_): δ = 15.3 (C25), 62.6 (C24), 101.7 (C9), 102.2 (C13), 113.7 (C12), 113.0 (C12), 114.2 (C20), 116.6 (C18), 118.4 (C1), 125.3 (C16), 125.5 (C5), 127.6 (C6), 129.3 (C13), 129.6 (C18), 133.2 (C10), 149.1 (C2), 153.8 (C14), 157.1 (C21), 159.7 (C7), 161.1 (C4). Calcd. for C_19_H_12_BrN_3_O_2_S (426.29) C, 53.53; H, 2.84; N, 9.86; S, 7.52 Found: C, 53.39; H, 2.75; Br, 18.68; N, 10.00; S, 7.41%.

### 3.5. Pyridine Derivatives ***10**–**14***

*l-(5-Bromobenzofuran-2-yl)-3-(dimethylamino)prop-2-en-l-one* (**3**). (1.86 g, 5 mmol), the appropriate acetylacetone, ethyl acetoacetate, ethyl cyanoacetate, malononitrile, benzoylacetonitrile, (5 mmol) and ammonium acetate (0.38 g, 5 mmol), was heated in acetic acid (10 mL) under reflux for 3 h. on cooling, the separated solid was filtered, washed with water and crystallized from the proper solvent afforded **10**–**14**, respectively.

*l-(6-(5-Bromobenzofuran-2-yl)-2-methylpyridin-3-yl)ethanone* (**10**). Beige crystals, Yield: 84%, melting point: 160–162 °C (acetic acid). IR (KBr, cm^−1^): 3001 (CH), 1710 (CO), 1648 (C=N), 1573 (C=C); ^1^H-NMR (400 MHz, DMSO-*d*_6_): δ = 2.51 (s, 3H, CH_3_), 2.57 (s, 3H, CH_3_), 7.31–7.89 (m, 6H, ArH’s); ^13^C*-*NMR (400 MHz, DMSO-*d*_6_): δ = 24.6 (C7), 28.5 (C9), 102.2 (C12), 114.1 (C19), 116.7 (C16), 118.8 (C1), 124.9 (C16), 129.0 (C13), 129.5 (C18), 131.2 (C6), 133.1 (C5), 152.2 (C1), 153.7 (C14), 158.0 (C4), 160.2 (C11), 201.1 (C8). MS, *m/z*, (%): 331 (M+1, 78%), 329 (M−1, 83%), 316 (100%), 314 (94%), 288 (16%), 286 (16%), 207 (48%), 204 (48%), 152 (18%), 150 (13%), 89 (25%), 77 (16%), 63 (36%); Calcd. for C_16_H_12_BrNO_2_ (330.18) C, 58.20; H, 3.66; Br, 24.20; N, 4.24 Found: C, 58.12; H, 3.58; Br, 24.00; N, 4.18%.

*Ethyl 6-(5-bromobenzofuran-2-yl)-2-methylpyridine-3-carboxylate* (**11**). Yellow crystals, Yield: 85%, melting point: 176–178 °C (dioxane). IR (KBr, cm^−1^): 3058 (CH), 1708 (CO), 1639 (C=N), 1585 (C=C); ^1^H-NMR (400 MHz, DMSO-*d*_6_): δ = 1.36 (t, 3H, *J* = 8.0 Hz, CH_2_CH_3_), 2.62 (s, 3H, CH_3_), 4.22 (q, 2H, *J* = 8.0 Hz, CH_2_CH_3_), 7.28–7.98 (m, 6H, ArH’s); MS, *m/z*, (%)): 361 (M+1, 64%), 359 (M−1, 100%), 317 (46%), 315 (63%), 259 (45%), 247 (63%), 89 (45%), 97 (45%), 62 (64%); ^13^C*-*NMR (400 MHz, DMSO-*d*_6_): δ = 14.2 (C22), 24.3 (C7), 61.8 (C22), 102.8 (C12), 114.0 (C19), 116.5 (C17), 120.1 (C1), 124.8 (C16), 125.0 (C5), 129.2 (C13), 129.7 (C18), 130.2 (C6), 148.8 (C2), 153.9 (C14), 157.0 (C4), 160.0 (C11), 166.8 (C8). Calcd. for C_17_H_14_BrNO_3_ (360.2) C, 56.69; H, 3.92; Br, 22.18; N, 3.89 Found: C, 56.58; H, 4.11; Br, 22.07; N, 3.96%.

*Ethyl 2-Amino-6-(5-bromobenzofuran-2-yl)pyridine-3-carboxylate* (**12**). Yellow crystals, Yield: 90%, melting point: 220–222 °C (dioxane). IR (KBr, cm^−1^): 3078 (CH), 1701 (CO), 1643 (C=N), 1550 (C=C); ^1^H-NMR (400 MHz, DMSO-*d*_6_): δ = 1.35 (t, 3H, *J* = 8.0 Hz, CH_2_CH_3_), 4.23 (q, 2H, *J* = 8.0 Hz, CH_2_CH_3_), 7.30–8.10 (m, 8H, NH_2_ and ArH’s); MS, *m/z*, (%): 362 (M+1, 53%), 360 (M−1, 50%), 290 (53%), 149 (53%), 90 (100%), 89 (53%), 81 (70%), 75 (47%); Calcd. for C_16_H_13_BrN_2_O_3_ (361.19) C, 53.21; H, 3.63; Br, 22.12; N, 7.76 Found: C, 53.27; H, 3.69; Br, 22.00; N, 7.68%.

*2-Amino-6-(5-bromobenzofuran-2-yl)pyridine-3-carbonitrile* (**13**). Brown crystals, Yield: 80%, melting point: 270–272 °C (dioxane). IR (KBr, cm^−1^): 3344, 3105 (NH_2_), 3078 (CH), 2218 (CN), 1653 (C=N), 1585 (C=C); ^1^H-NMR (400 MHz, DMSO-*d*_6_): δ = 6.21 (s, br., 2H, NH_2_), 7.30–8.22 (m, 6H, ArH’s); MS, *m/z*, (%): 315 (M+1, 94%), 313 (M−1, 100%), 289 (11%), 287 (12%), 164 (11%), 129 (11%), 127 (16%), 75 (25%); Calcd. for C_14_H_8_BrN_3_O (314.14) C, 53.53; H, 2.57; Br, 25.44; N, 13.38 Found: C, 53.48; H, 2.61; Br, 25.33; N, 13.29%.

*(2-Amino-6-(5-bromobenzofuran-2-yl)pyridin-3yl)(phenyl)methanone* (**14**). Brown crystals, Yield: 90%, melting point: 240–242 °C (acetic acid). IR (KBr, cm^−1^): 3344, 3105 (NH_2_), 3078 (CH), 1680 (CO), 1624 (C=N), 1577 (C=C); ^1^H-NMR (400 MHz, DMSO-*d*_6_): δ = 7.30–7.79 (m, 11H, ArH’s), 10.21 (s, br., 2H, NH_2_); MS, *m/z*, (%): 394 (M+1, 77%), 393 (M^+^, 54%), 392 (17%), 290 (33), 288 (35%), 224 (68%), 222 (67%), 168 (17%), 166 (52%), 146 (15%), 144 (17%), 109 (56%), 88 (97%), 75 (31%); Calcd. for C_20_H_13_BrN_2_O_2_ (393.23) C, 61.09; H, 3.33; Br, 20.32; N, 7.12 Found: C, 61.15; H, 3.42; Br, 20.12; N, 7.00%.

*6-(5-Bromobenzofuran-2-yl)-2-methylpyridine-3-carbohydrazide* (**15**). A mixture of **12** (1.85 g, 5 mmol) and hydrazine hydrate (1 g, 20 mmol) in ethanol (20 mL) was heated under refluxed for 3 h. The resulting solid was collected and recrystallized from acetic acid gave a beige crystals. Yield: 96%, melting point: 250–252 °C. IR (KBr, cm^−1^): 3388, 3337, 3217 (NH, NH_2_), 3062 (CH), 2920, 2851 (CH), 1680 (CO), 1640 (C=N), 1589 (C=C); ^1^H-NMR (400 MHz, DMSO-*d*_6_): δ = 2.62 (s, 3H, CH_3_), 6.24 (s, br., 3H, NH and NH_2_), 7.23–7.89 (m, 6H, ArH’s); MS, *m/z*, (%): 347 (M+1, 15%), 345 (M−1, 13%), 315 (79%), 314 (100%), 313 (86%), 207 (43%), 205 (40%), 152 (20%), 151 (23%), 150 (0%), 103 (18%), 77 (25%), 63 (43%); Calcd. for C_15_H_12_BrN_3_O_2_ (346.18) C, 52.04; H, 3.49; Br, 23.08; N, 12.14 Found: C, 52.04; H, 3.49; Br, 23.08; N, 12.14%

### 3.6. 1-(6-(5-Bromobenzofuran-2-yl)-2-methylnicotinoyl)-3-methyl-1H-pyrazol-5(4H)-one *(**16a**)* and (6-(5-Bromobenzofuran-2-yl)-2-methylpyridin-3-yl)(3,5-dimethyl-1H-pyrazol-1-yl)methanone *(**16b**)*

A mixture of 6-(5-bromobenzofuran-2-yl)-2-methylpyridine-3-carbohydrazide (**15**) (1.73 g, 5 mmol), ethyl acetoacetate or acetylacetone in ethanol (20 mL) and acetic acid (5 drops) was heated under reflux for 3 h. on cooling, the separated yellow solid was filtered, washed with water and crystallized gave **16a** and **16b**, respectively.

*1-(6-(5-Bromobenzofuran-2-yl)-2-methylnicotinoyl)-3-methyl-1H-pyrazol-5(4H)-one* (**16a**). Yellow crystals, Yield: 87%, melting point: 260–262 °C (DMF). IR (KBr, cm^−1^): 2920 (CH), 1687 (CO), 1639 (C=N), 1589 (C=C); ^1^H*-*NMR (400 MHz, DMSO-*d*_6_): δ = 2.10 (s, 3H, CH_3_), 2.64 (s, 3H, CH_3_), 3.42 (q, 1H, CH_2_), 3.62 (q, 1H, CH_2_), 7.30–7.95 (m, 6H, ArH’s); MS, *m/z*, (%): 413 (M+1, 19%), 411 (M−1, 18%), 98 (48%), 91 (22%), 88 (44%), 86 (30%), 80 (85%), 64 (44%); Calcd. for C_19_H_14_BrN_3_O_3_ (412.24) C, 55.36; H, 3.42; Br, 19.38; N, 10.19 Found: C, 55.41; H, 3.38; Br, 19.28; N, 10.00%.

*(6-(5-Bromobenzofuran-2-yl)-2-methylpyridin-3-yl)(3,5-dimethyl-1H-pyrazol-1-yl)methanone* (**16b**). Yellow crystals, Yield: 91%, melting point: 272–274 °C (dioxan). IR (KBr, cm^−1^): 2977 (CH), 1681 (CO), 1585 (OC); ^1^H-NMR (400 MHz, DMSO-*d*_6_): δ = 2.30 (s, 3H, CH_3_), 2.36 (s, 3H, CH_3_), 2.66 (s, 3H, CH_3_), 5.78 (s, 1H, pyrazole H-4), 7.30–7.99 (m, 6H, ArH’s); MS, *m/z*, (%): 410 (M^+^, 100%), 331 (48%), 316 (5%), 314 (8%), 289 (10%), 206 (16%), 179 (49%), 167 (16%), 165 (11%), 139 (11%), 137 (11%), 113 (15%), 111 (19%), 91 (35%), 77 (34%), 65 (12%); Calcd. for C_20_H_16_BrN_3_O_2_ (410.26) C, 58.55; H, 3.93; Br, 19.48; N, 10.24 Found: C, 58.48; H, 4.12; Br, 19.52; N, 10.00%.

### 3.7. 2-[6-(5-Bromobenzofuran-2-yl)-2-methyl-pyridine-3-carbonyl]-5-methyl-4-(phenyl-hydrazono)-2,4-dihydro-pyrazol-3-one *(**17**)* and (6-(5-bromobenzofuran-2-yl)-2-methylpyridin-3-yl)(3,5-dimethyl-4-(2-phenylhydrazinyl)-1H-pyrazol-1-yl)methanone *(**18**)*

***Method A***: benzenediazonium chloride (5 mmol), which was prepared *via* reaction of aniline (0.46 g. 5 mmol), hydrochloric acid (3 mL, 6 M) and sodium nitrite (0.37 gm, 5 mniole) at 0–5 °C, was added to a mixture of the appropriate **16a** or **16b** (5 mmole) and sodium acetate (0.41 gm, 5 mmole) in ethanol (30 mL) at 0–5 °C, while stirring. The reaction mixture was stilted for 3 h. The resulting solid, was collected, washed with water and recrystallized from acetic acid gave **17** and **18**, respectively.

***Method B***: A mixture of **15** (1.73 g, 5 mmol) and the appropriate of ethyl 2-(2-phenylhydrazono)-3-oxobutanoate (**19a**) or 3-(2-phenyl-hydrazono)pentane-2,4-dione (**19b**) (5 mmol) in ethanol (20 mL) and catalytic amount of acetic acid (2 drops) was refluxed for 2 h. The resulting solid, so formed, was collected and recrystallized from acetic acid gave products identical in all aspects to those obtained from method A.

*2-[6-(5-Bromobenzofuran-2-yl)-2-methyl-pyridine-3-carbonyl]-5-methyl-4-(pheny1-hydrazono)-2,4-dihydro-pyrazoI-3-one* (**17**). Brown crystals, Yield: 82%, melting point: 276–278 °C (DMF). IR (KBr, cm^−1^): 3345 (NH), 2989 (CH), 1712 (CO), 1639 (C=N), 1581 (C=C); ^1^H*-*NMR (400 MHz, DMSO-*d*_6_): δ = 2.12 (s, 3H, CH_3_), 2.68 (s, 3H, CH_3_), 7.11–7.99 (m, 11H, ArH’s), 10.88 (s, br., 1H, NH); MS, *m/z*, (%): 516 (M^+^, 13%), 423 (39%), 420 (13%), 394 (10%), 392 (75%), 362 (25%), 346 (17%), 318 (9%), 316 (15%), 290 (11%), 288 (9%), 195 (19%), 193 (19%), 167 (17%), 165 (11%), 139 (100%), 114 (22%), 112 (35%), 100 (37%), 87 (41%), 75 (57%), 62 (31%); Calcd. for C_25_H_18_BrN_5_O_3_ (516.35) C, 58.15; H, 3.51; Br, 15.47; N, 13.56 Found: C, 58.08; H, 3.64; Br, 15.52; N, 13.61%.

*(6-(5-Bromobenzofuran-2-yl)-2-methylpyridin-3-yl)(3,5-dimethyl-4-(2-phenylhydrazinyl)-1H-pyrazol-1-yl)methanone* (**18**). Brown crystals, Yield: 82%, melting point: 230–232 °C (DMF). IR (KBr, cm^−1^): 2916 (CH), 1652 (CO), 1616 (C=N), 1546 (C=C); ^1^H-NMR (400 MHz, DMSO-*d*_6_): δ = 2.18 (s, 3H, CH_3_), 2.62 (s, 3H, CH_3_), 2.68 (s, 3H, CH_3_), 7.31–8.35 (m, 11H, ArH’s); MS, *m/z*, (%): 515 (M+1, 0.98%), 513 (M−1, 75%), 223 (13%), 252 (13%), 251 (11%), 213 (15%), 211 (14%), 169 (6%), 167 (7%), 116 (35%), 114 (28%), 102 (27%), 87 (85%), 77 (50%), 62 (100%); Calcd. for C_26_H_20_BrN_5_O_2_ (514.37) C, 60.71; H, 3.92; Br, 15.53; N, 13.62 Found: C, 60.64; H, 4.10; Br, 15.39; N, 13.52%.

*6-(5-Bromobenzofuran-2-yl)-2-methylnicotinoyl azide* (**20**). A stirred solution of **15** (1.78 g, 5 mmol) in hydrochloric acid (15 mL, 6M) at 0-5 °C, sodium nitrite was added portion-wise tell effervescence ended. The reaction mixture was stirred for 1 h. The resulting solid, was collected, filtered, washed with water and recrystallized from DMF gave a beige crystals. Yield: 78%, melting point: >300 °C. IR (KBr, cm^−1^): 3070(CH), 2989, 2927 (CH), 2124 (Azide), 1712 (CO), 1639 (C=N), 1581 (OC); ^1^H-NMR (400 MHz, DMSO-*d*_6_): δ = 2.61 (s, 3H, CH_3_), 7.12–7.95 (m, 6H, ArH’s); MS, *m/z*, (%): 359 (M+2, 5%), 357 (M^+^, 6%), 330 (89%), 328 (87%), 304 (92%), 302 (100%), 223 (16%), 221 (15%), 194 (14%), 192 (14%), 180 (15%), 178 (13%), 152 (33%), 150 (27%), 126 (23%), 124 (13%), 113 (16%), 97 (32%), 77 (42%), 62 (55%); Calcd. for C_15_H_9_BrN_4_O_2_ (357.16) C, 50.44; H, 2.54; Br, 22.37; N, 15.69 Found: C, 50.38; H, 2.47; Br, 22.42; N, 15.75%.

### 3.8. Urea Derivatives ***21a**–**e***

A mixture of appropriate aniline, *p*-toluidine, *p*-anisidine, 3-amino-5-phenylpyrazole or 3-amino-l,2,4-triazole (5 mmol) and azido compound **20** (1.78 g, 5 mmol) in dry dioxane (20 mL) was refluxed for 4 h. The resulting solid, so formed, was collected and recrystallized gave **21a**–**d**, respectively.

*1-(6-(5-Bromobenzofuran-2-yl)-2-methylpyridin-3-yl)-3-phenylurea* (**21a**). Yellow crystals. Yield: 94%, melting point: 268–270 °C (DMF). IR (KBr, cm^−1^): 3103 (NH), 3055 (CH), 2920, 2850 (CH), 1700 (CO), 1639 (C=N), 1589 (OC); ^1^H-NMR (400 MHz, DMSO-*d*_6_): δ = 2.15 (s, 3H, CH_3_), 7.00–7.95 (m, 11H, ArH’s), 8.67 (s, br., 2H, 2NH); MS, *m/z*, (%): 422 (M^+^,5%), 420 (5%), 213 (8%), 151 (9%), 119 (13%), 116 (29%), 1–14 (14%), 90 (18%), 87 (60%), 77 (72%), 62 (100%); Calcd. for C_21_H_16_BrN_3_O_2_ (422.27) C, 59.73; H, 3.82; Br, 18.92; N, 9.95 Found: C, 59.69; H, 3.88; Br, 19.12; N, 10.00%.

*l-(6-(5-Bromobenzofuran-2-yl)-2-methylpyridin-3-yl)-3-p-tolylurea* (**21b**). White crystals. Yield: 93%, melting point: 290–292 °C (DMF). IR (KBr, cm^−1^) 255 (NH), 3070 (CH), 2916, 2850 (CH), 1690 (CO), 1639 (C=N), 1593 (C=C); ^1^H-NMR (400 MHz, DMSO-*d*_6_): δ = 2.10 (s, 3H, CH_3_), 2.24 (s, 3H, CH_3_), 7.00–7.66 (m, 10H, ArH’s), 8.75 (s, br., 2H, 2NH); MS, *m/z*, (%): 438 (M+2, 5%), 436 (M^+^, 5%), 304 (13%), 169 (12%), 167 (11%), 106 (27%), 88 (17%), 87 (31%), 86 (28%), 77 (26%), 62 (100%); Calcd. for C_22_H_18_BrN_3_O_2_ (436.3) C, 60.56; H, 4.16; Br, 18.31; N, 9.63 Found: C, 60.56; H, 4.16; Br, 18.31; N, 9.63%.

*l-(6-(5-Bromobenzofuran-2-y1)-2-methylpyridin-3-yl)-3-(4-methoxyphenyl) urea* (**21c**). Beige crystals. Yield: 92%, melting point: 280–252 °C (DMF). IR (KBr, cm^−1^): 3255 (NH), 3070 (CH), 2916, 2850 (CH), 1690 (CO), 1639 (C=N),1593 (C=C); ^1^H-NMR(400 MHz, DMSO-d_6_): δ = 2.10 (s, 3H, CH_3_), 3.71 (s, 3H, CH_3_), 6.87–8.52 (m, 10H, ArH’s), 9.25 (s, br., 2H, 2NH); MS, *m/z*, (%): 452 (M^+^, 1.3%), 451 (4%), 333 (7%), 332 (20%), 331 (100%), 238 (54%), 175 (12%), 160 (64%), 155 (62%), 93 (35%), 91 (54%), 84 (17%); Calcd. for C_22_H_18_BrN_3_O_3_ (452.3) C, 58.42; H, 4.01; Br, 17.67; N, 9.29 Found: C, 58.48; H, 4.11; Br, 17.71; N, 9.34%.

*1-(6-(5-Bromobenzofuran-2-yl)-2-methylpyridin-3-yl)-3-(3-phenyl-1H-pyrazol-5-yl)urea* (**21d**). Yellow crystals. Yield: 92%, melting point: 262–264 °C (DMF). IR (KBr, cm^−1^): 3101 (NH), 3058 (CH), 2916, 2850 (CH), 1690 (CO), 1639 (C=N), 1589 (C=C); ^1^H-NMR (400 MHz, DMSC-d_6_): δ = 2.11 (s, 3H, CH_3_), 5.34 (s, 1H, pyrazole H-4), 7.22–7.79 (m, 11H, ArH’s), 9.88 (s, br., 3H, 3NH); Calcd. for C_24_H_18_BrN_5_O_2_ (488.34) C, 59.03; H, 3.72; Br, 16.36; N, 14.34 Found: C, 59.03; H, 3.72; Br, 16.36; N, 14.34%.

*1-(6-(5-Bromobenzofuran-2-yl)-2-methylpyridin-3-yl)-3-(4H-1,2,4-triazol-3-yl)urea* (**21e**). Yellow crystals. Yield: 93%, melting point: 274–276 °C (DMF). IR (KBr, cm^−1^): 3101 (NH), 3058 (CH), 2916, 2850 (CH), 1690 (CO), 1639 (C=N), 1589 (OC); ^1^H-NMR (400 MHz, DMSO-*d*_6_): δ = 2.10 (s, 3H, CH_3_), 7.22–7.79 (m, 10H, ArH’s), 9.897 (s, br., 3H, 3NH); Calcd. for C_17_H_13_BrN_6_O_2_(413.23) C, 49.41; H, 3.17; Br, 19.34; N, 20.34 Found: C, 49.38; H, 3.21; Br, 19.29; N, 20.41%.

*3-(6-(5-Bromobenzofuran-2-yl)-2-methylpyridin-3-yl)quinazoline-2,4(1H,3H)-dione* (**22**). A mixture of appropriate methyl anthranilate or anthranilic acid (5 mmol) and azido compound **20** (1.78 g, 5 mmol) in dry dioxane (20 mL) was refluxed for 4 h. The resulting solid, so formed, was collected and recrystallized from DMF gave **22** as beige crystals Yield: 87.6%, melting point: >300 °C. IR (KBr, cm^−1^): 3255 (NH), 3062 (CH), 2923 (CH), 1681 (CO), 1639 (C=N), 1589 (C=C); ^1^H-NMR (400 MHz, DMSO-*d*_6_): δ = 2.48 (s, 3H, CH_3_), 7.14–8.22 (m, 10H, ArH’s), 10.55 (s, br., 1H, NH); ^13^C*-*NMR (400 MHz, DMSO-*d*_6_): δ = 21.2 (C8), 102.4 (C21), 114.1 (C13), 114.2 (C28), 115.1 (C17), 126.4 (C26), 122.2 (C1), 123.2 (C19), 125.0 (C25), 127.0 (C20), 129.3 (C22), 129.7 (C27), 131.0 (C6), 135.0 (C18), 138.1 (C5), 148.1 (C2), 149.0 (C10), 154.0 (C13), 158.0 (C4), 159.6 (C7), 163.1 (C14). Calcd. for C_22_H_14_BrN_3_O_3_ (448.27) C, 58.95; H, 3.15; Br, 17.83; N, 9.37 Found: C, 59.12; H, 3.04; Br, 17.75; N, 9.3742%.

*Phenyl 6-(5-bromobenzofuran-2-yl)-2-methylpyridin-3-ylcarbamate* (**23**). A mixture of **20** (1.78 g, 5 mmol) and phenol (5 mmol) in dry benzene (20 mL) was refluxed for 4 h. The resulting solid, so formed, was collected and recrystallized from dioxane to give **23** as beige crystals Yield: 87.6%, melting point: >300 °C. IR (KBr, cm^−1^): 3255 (NH), 3062 (CH), 2923 (CH), 1670 (CO), 1620 (C=N), 1566 (C=C); ^1^H-NMR (400 MHz, DMSO-*d*_6_): δ = 2.48 (s, 3H, CH_3_), 7.14–8.22 (m, 11H, ArH’s), 10.55 (s, br., 1H, NH); Calcd. for C_21_H_15_BrN_2_O_3_(423.26) C, 59.59; H, 3.57; Br, 18.88; N, 6.62 Found C, 59.64; H, 3.59; Br, 18.75; N, 6.57%.

## 4. Conclusions

Compound **1** proved to be a useful precursor for synthesis of various pyridines and thieno[2,3-*b*]pyridines via its reactions with the appropriate cyanothioacetamide, 2-cyanoacetohydrazidem, pentane-2,4-dione, ethyl 3-oxobutanoate, ethyl cyanoacetate or benzoylacetonitrile. Moreover, compound **15** proved a useful precursor in the synthesis of various urea and carbomate derivatives. The structures of the newly synthesized compounds were confirmed by spectral data and elemental analyses.
